# A Humidity Sensing Organic-Inorganic Composite for Environmental Monitoring

**DOI:** 10.3390/s130303615

**Published:** 2013-03-14

**Authors:** Zubair Ahmad, Qayyum Zafar, Khaulah Sulaiman, Rizwan Akram, Khasan S. Karimov

**Affiliations:** 1 Low Dimensional Materials Research Centre, Department of Physics, University of Malaya, Kuala Lumpur 50603, Malaysia; E-Mail: qayyumzafar@gmail.com (Q.Z.); khaulah@um.edu.my (K.S.); 2 Department of Electrical Engineering, College of Engineering, Qassim University, P.O. Box 6688 Qassim 51452, Saudi Arabia; E-Mail: rizwanakram75@qec.edu.sa; 3 Ghulam Ishaq Khan Institute of Engineering Sciences and Technology, Topi 23640, Pakistan; E-Mail: khasan@giki.edu.pk

**Keywords:** composite film, humidity sensor, physical vapor deposition, scanning electron micrograph

## Abstract

In this paper, we present the effect of varying humidity levels on the electrical parameters and the multi frequency response of the electrical parameters of an organic-inorganic composite (PEPC+NiPc+Cu_2_O)-based humidity sensor. Silver thin films (thickness ∼200 nm) were primarily deposited on plasma cleaned glass substrates by the physical vapor deposition (PVD) technique. A pair of rectangular silver electrodes was formed by patterning silver film through standard optical lithography technique. An active layer of organic-inorganic composite for humidity sensing was later spun coated to cover the separation between the silver electrodes. The electrical characterization of the sensor was performed as a function of relative humidity levels and frequency of the AC input signal. The sensor showed reversible changes in its capacitance with variations in humidity level. The maximum sensitivity ∼31.6 pF/%RH at 100 Hz in capacitive mode of operation has been attained. The aim of this study was to increase the sensitivity of the previously reported humidity sensors using PEPC and NiPc, which has been successfully achieved.

## 1. Introduction

Despite the current applications of inorganic materials in the fabrication of sensors, the market is demanding economical and miniaturized sensors. Organic semiconductors are materials that can be deposited at low temperature through uncomplicated deposition methods like spin coating and drop casting. These features give organic semiconductors an economic and technological edge over conventional inorganic semiconductor sensing materials. Consequently, extensive R&D efforts have been directed towards various organic semiconductors and their composites to identify appropriate materials for humidity sensing applications [1–5]. A good sensing material must meet certain stringent requirements such as high sensitivity, reproducibility, fast response and recovery time, small hysteresis, low power consumption and portability, along with durability and long lifetime [6].

In the past few decades, conventional inorganic semiconductors, particularly porous silicon, have been extensively utilized for humidity sensing applications. The working principle of these sensors is the change in conductivity of the humidity sensing layer. However, problems associated with conventional humidity sensors are the considerably higher cost of pure base materials and the high temperature fabrication procedures needed [7–9]. Organic materials are very attractive from the point of view of their low cost production. The preparation of organic sensitive layers is done by economical and uncomplicated wet deposition procedures which usually involve spin coating, drop casting, dip coating, or spray coating directly from liquid solutions. However, in comparison to conventional materials, organic materials show relatively worse long term mechanical and electrical parameter stability. Still this drawback of organic semiconductors doesn't limit their application in economical disposable sensor technology, as they can tolerate stability issues.

Based on the operating principle, organic material based humidity sensors fall into various categories *viz.*, capacitive, resistive, hydrometric, gravimetric, optical, and integrated types [10–12]. The mode of operation for the capacitive type sensors is a change in dielectric constant of the humidity sensing layer with varying humidity levels. Contrariwise, a resistive humidity sensor works on the resistance-variation principle. Owing to their advantages, including low power consumption and large output signal [13], roughly 75% of the sensors being employed in the commercial market are capacitive-type humidity sensors [11]. The performance of capacitive and resistive sensors is primarily dependent upon the properties of the hygroscopic material used to fabricate the sensing film and secondly, the design of the sensor electrodes [14].

Nickel phthalocynine (NiPc), copper oxide (Cu_2_O), poly-*N*-epoxypropylcarbazole (PEPC) and mixtures of the latter two have already been reported as ambient sensitive organic semiconductors. The humidity sensing capability of a blend of Cu_2_O and PEPC has been investigated in our previous study and the bandwidth of the sensor was found to be 36-86% RH, whilst its sensitivity was found to be 0.83 pF/%RH [3]. The humidity sensing capability of NiPc has also been investigated earlier by Shah *et al.* [1] who reported a nonlinear increase in capacitance value with increasing humidity level from 35% to 90% RH, while the sensitivity was reported to be 8.93 pF/%RH. Keeping in mind the potential of NiPc and PEPC for humidity sensors, we have investigated a film of organic-inorganic composite of PEPC, NiPC and Cu_2_O as humidity sensor, where Cu_2_O is used as template to enhance the surface roughness. PEPC and NiPc create charge-transfer complexes due to the partial transfer of electrons from PEPC into NiPc. Due to the formation of the PEPC-NiPc complexs the resistance of the composite also decreases, that allows the capacitance of the humidity sensor to increase. The purpose of this study is to tune the sensitivity, bandwidth and linearity in response characteristics of the humidity sensor, with the aim of increasing the sensitivity of the previously reported humidity sensors using PEPC and NiPc [1,15,16], which has been successfully achieved.

## 2. Experimental

**Figure 1.**
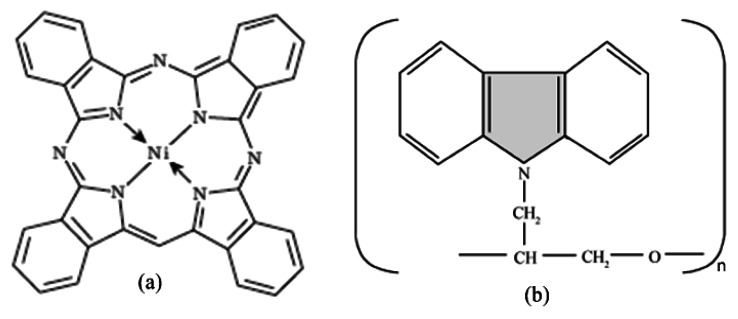
(**a**) Molecular structure of nickel phthalocyanine (NiPc); (**b**) molecular structure of poly-N-epoxypropylcarbazole (PEPC).

**Figure 2.**
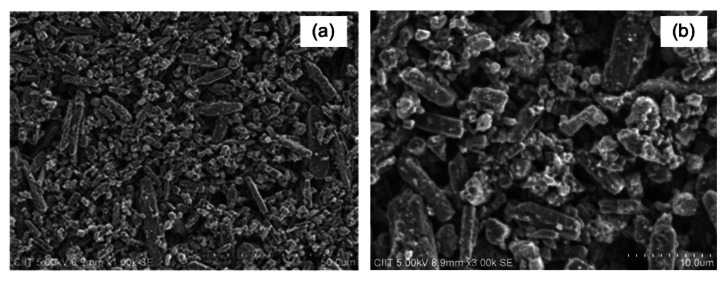
(**a**) and (**b**) represent SEM micrographs of Cu_2_O-PEPC-NiPC thin film at 1K and 3K magnification, respectively.

**Figure 3.**
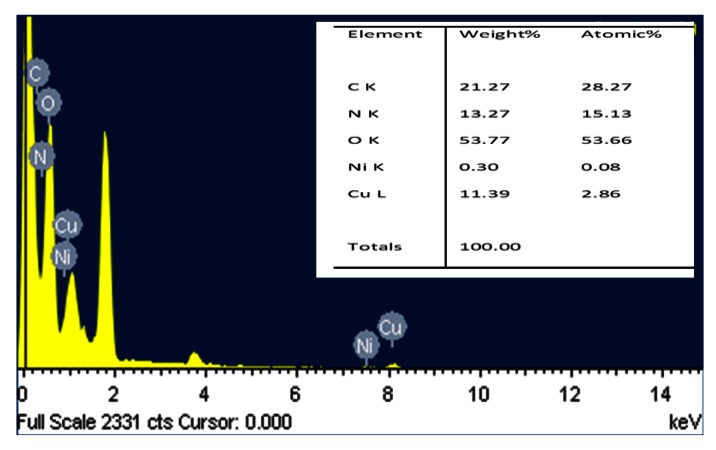
EDX compositional analysis of organic-inorganic composite film.

Thin films of organic-inorganic composite, Cu_2_O-PEPC-NiPC serve as an active sensing layer in the fabrication of Ag/Cu_2_O-PEPC-NiPC/Ag surface type humidity sensors. Commercially available Cu_2_O microparticles, having a diameter in the range of 3–4 μm, were purchased from WINLAB UK (Leicestershire, UK), whereas nickel phthalocyanine powder was purchased from Sigma Aldrich (Lahore, Pakistan), and was used without further purification. PEPC however was synthesized in the laboratory, and its fabrication process has been described in detail elsewhere [17]. Figures 1(a,b) show the molecular structures of nickel phthalocyanine and PEPC. Figures 2(a,b) show SEM micrographs of thin films of Cu_2_O-PEPC-NiPC, obtained using a Hitachi SU-1500 scanning electron microscope (Hitachi High-Technologies, Tokyo, Japan). The SEM micrographs show porosity in the structure, suggesting Cu_2_O-PEPC-NiPC thin film is ideal for humidity sensing studies. Figure 3 shows an EDX graph showing elemental ratios of C, N, O, Ni and Cu. As is known, EDX is unable to determine the elemental ratio of hydrogen, so the elemental ratio of hydrogen in the composite is not mentioned.

Seven weight% Cu_2_O, 4 weight% PEPC and 5 weight% NiPC were initially blended in Benzol (Cyclohexa-1,3,5-triene, IUPAC name; Benzene). Unlike PEPC and NiPC, Cu_2_O is inorganic in nature and is not dissolved in the organic solvent Benzene. This results in a colloidal solution of Cu_2_O-PEPC-NiPC composite in Benzene. A thin film of this suspension served as a sensing layer in our fabricated humidity sensor. Commercially available glass slides, which were thoroughly cleaned ultrasonically with distilled water for 10 minutes and later dried in a dust free environment, were utilized as a substrate. Glass slides were further plasma cleaned for 5 minutes with the subsequent fabrication step. Thin films of silver (thickness ∼200 nm) were deposited on a glass substrate by a thermal evaporator. An Edward Auto 306 vacuum coater with a diffusion pumping system (Edwards High Vacuum, Sussex, UK) was used, which also provided the facility of plasma cleaning of the substrate's surface. While depositing a silver thin film, the pressure inside the chamber was kept at 10^−5^ mbar, whereas the deposition rate was maintained at 0.1 nm/s.

**Figure 4.**
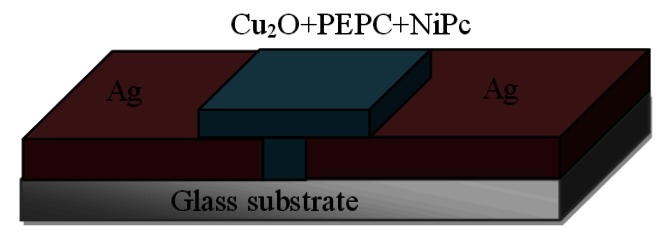
Schematic view of Ag/Cu_2_O-PEPC-NiPC/Ag humidity sensor.

Silver thin films were later patterned by using standard optical lithographic techniques, thus producing a pair of silver electrodes on the glass substrate. The silver thin film was etched by wet etching, using liquid nitric acid (HNO_3_). The gap between the surface type silver electrodes was 20 μm, which was subsequently covered by solution of organic-inorganic composite Cu_2_O-PEPC-NiPC in Benzene. The active sensing layer of Cu_2_O-PEPC-NiPC with thickness of 3–5 μm (the results presented here are the average results for 4 μm thick film) and the surface area 4 mm × 4 mm, was obtained by the spin coating technique and was dried under ambient conditions. A schematic view of the fabricated Ag/Cu_2_O-PEPC-NiPC/Ag surface type sensor is shown in Figure 4.

Silver was selected for the electrode formation since its adhesion to glass substrates is pretty good and is not affected by repeated exposure of the sensor to humidity. Consequently we find extensive application of silver electrodes in the fabrication of sensors [8,18,19].

**Figure 5.**
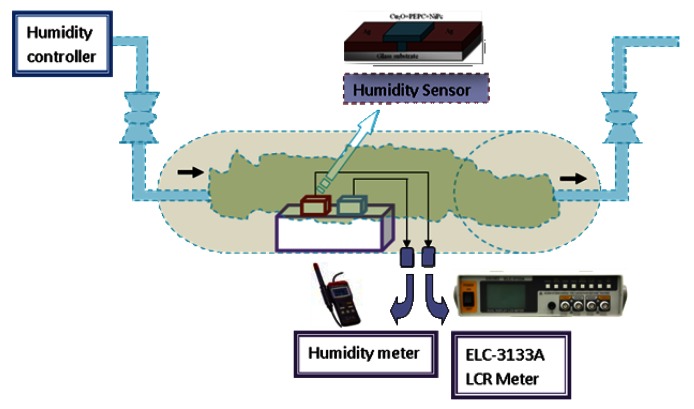
Characterization setup for electrical characterization of the humidity sensor.

The humidity sensor was subjected to electrical characterization in a sealed chamber with a controlled humidity environment. The chamber was fabricated in our device testing laboratory and it had inlet and outlet valves for gas flow. To create humidity in the chamber, nitrogen gas is passed through water and then injected into the chamber. Monitoring of temperature and humidity levels in the humidity chamber was done by a humidity meter (Mastech MS 6503, Dongguan Huayi Mastech Company Limited, Dongguan, China). The capacitance of the sensor was measured by Escort ELC 3133A Bench LCR Meter (Instruments Techno Test inc, Laval, QC, Canada). The root mean square value of the applied voltage (V_RMS_) for the AC measurements of sensor was 1.0 V. The frequency response of the sensor was studied by the recording capacitance of the sensor over a wide range of operating frequency, *i.e.*, 100 Hz, 1 kHz and 10 kHz. The characterization setup has been portrayed in Figure 5. Humidity level monitoring by the humidity meter was prone to an instrumental error of ±1% RH. The repeatability of the measurements was checked six times and was found within ±5.3%. All measurements were taken at 25 °C.

## 3. Results and Discussion

**Figure 6.**
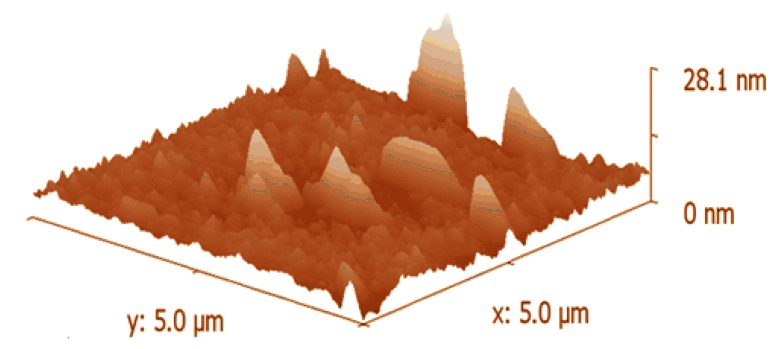
3D AFM micrograph of organic-inorganic composite film.

Figure 6 shows the 3D AFM micrograph, obtained in tapping mode at room temperature, with a scan size area of 5 μm.

This AFM image suggests a rough morphology of the active sensing layer. It is well understood, that there exists a direct relationship between the morphology of the semiconductor sensing layer and the absorption of water molecules. The rougher the surface the greater the number of adsorption sites that would be available for water molecules [2].

**Figure 7.**
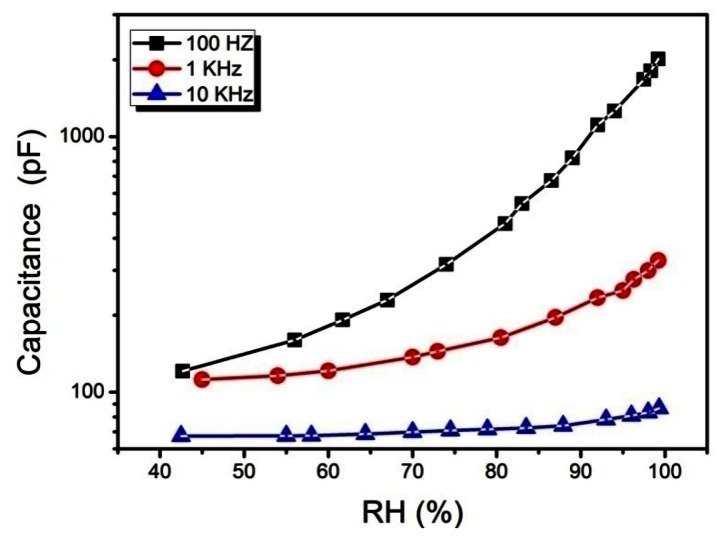
Capacitance-relative humidity relationship of the Ag/Cu_2_O-PEPC-NiPC/Ag sensor at 100 Hz, 1 kHz and 10 kHz.

Figure 7 shows capacitance-relative humidity relationship, for the Ag/Cu_2_O-PEPC-NiPC/Ag humidity sensor. It is observed from the experimental results that capacitance shows positive deviation in its value from 134 pF to 2,137 pF when the relative humidity increases from 40% to 100% RH.

The changes in capacitance as a function of relative humidity, at three distinct AC frequencies of applied voltage *i.e.*, 100 Hz, 1 kHz and 10 kHz, were found to be 16 times, 3.4 times, and 1.36 times, respectively. The change in capacitance of the sensor as a function of humidity is associated with the porosity and surface roughness of the organic thin film. It may be suggested that rougher and porous surfaces ensure the availability of more absorption sites for water molecules. Further, with increasing humidity levels, absorption of water molecules by the active sensing film increases progressively and a significant change in capacitance of the sensor is thus observed. The organic active thin film is used as dielectric which adsorbs or desorbs water molecules proportional to the relative environmental humidity, and thus changes the capacitance of the capacitor (Ag/Cu_2_O-PEPC-NiPC/Ag). The capacitive humidity sensors, typically exhibit a non-linear response as a function of relative humidity. The reason of change in capacitance with RH may be attributed to the immense difference in the dielectric values of water and composite film. At 25 °C, dielectric constant of water is ∼80, however organic semiconductors generally have small dielectric values. With the increase in absorption of water molecules, water molecule layer gets stacked up on composite layer, changing the net dielectric permittivity of the film. Dielectric constant thus plays pivotal role in the variation of capacitance, as both are directly related to each other. The relation between dielectric constant and capacitance can be described by the following equation [14]:

(1) $$\frac{C_{S}}{C_{0}}={\left(\frac{{ɛ}_{w}}{{ɛ}_{d}}\right)}^{n},$$

where *ε_d_*, and *ε_w_* are the dielectric permittivities of the composite film at dry and wet conditions, respectively. “*n*” is morphological factor.

Variation in capacitance is also associated with the polarization of the composite film [20]. Polarization may arise in the composite film due to formation of ions and dipoles. Particularly ions in inorganic ionic compounds like Cu_2_O, and dipoles are formed in organic π-conjugated large molecules and polymeric semiconductor molecule, due to charge transfer complexes and dissociation of molecules due to the introduction of water contents in the composite film. Consequently it can be assumed that capacitance increases due to polarization in composite film and dipole formation (H^+^, HO^−^) in water molecules.

As it is well understood that organic semiconductors are unsuitable for high frequency electronics applications such as high frequency switching devices [21]. Hence our findings have strong correlation with the aforementioned fact, as our sensor provides maximum sensitivity at lower frequency. Since the dipoles of π-conjugated large organic molecules have lower mobility, thus at higher frequencies they have insufficient time to respond to the behavior of the applied AC signal [22], thereby reducing the sensitivity at higher order of frequency.

**Figure 8.**
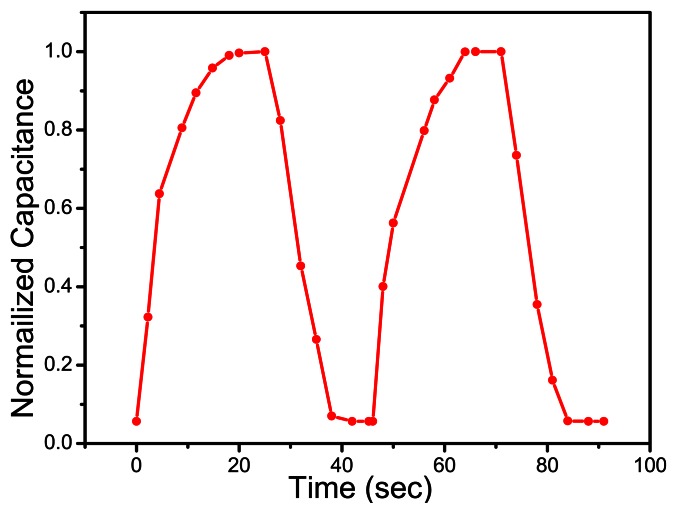
Response and recovery time (40% RH-97% RH level) of the Ag/Cu_2_O-PEPC-NiPC/Ag humidity sensors at 100 Hz.

The response and recovery time is an important characteristic for evaluating the performance of humidity sensors. The response time can be defined as the time required for a sensor output to change from its previous state to 90% of its final settled value. The recovery time of a sensor is reported as the time taken from the output of sensor to fall to 10% of final settled value. Step introduction and removal of measured variable, which in our case was relative humidity, is vital for response/recovery measurement. As portrayed in Figure 8, when the humidity level was increased from 40 to 97%, the response time for our sensor was 13 seconds and when the RH was decreased from 97 to 40%, the recovery time was 15 seconds.

**Figure 9.**
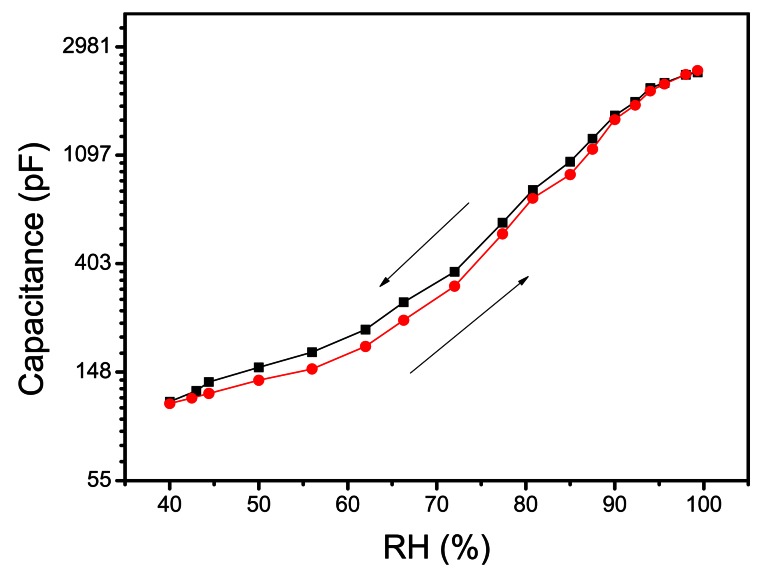
Hysteresis-relative humidity relationship for Ag/Cu_2_O-PEPC-NiPC/Ag.

One of the most important challenges with humidity sensors based on the principle of absorption is hysteresis. Hysteresis is defined as the percentage difference in final settling point of relative humidity when approached from above to when it is approached from below. This phenomenon exists due to the formation of clusters of absorbed water in the active thin film and it depends upon the geometry of the pores. The larger pore size reduces the response time but they also reduce the sensitivity so it is difficult to make fast and sensitive humidity sensor. In our study, the hysteresis property of humidity sensor was studied by measuring capacitance at 100 Hz AC signal. Experimental data (Figure 9) showed that device had 12% hysteresis. The hysteresis study of the sensor was repeated after a month's time with 10 cycles a day, which showed that the average value of hysteresis increased from 12% to 13.3%.

## 4. Conclusions

In this study, an organic-inorganic composite-based humidity sensor Ag/Cu_2_O-PEPC-NiPC/Ag, was fabricated in planar configuration and its sensing performances were investigated experimentally, which included measurement of the capacitance of the sensor as a function of different environmental humidity levels. The sensor has been found to have good parameter stability and adequate sensorial properties. In general an increase in capacitance was observed with increasing humidity from 40% RH to 100% RH. Furthermore, as the composite film has earlier been found to be pressure sensitive as well [23], the fabrication of the Ag/Cu_2_O-PEPC-NiPC/Ag surface type humidity sensor would also help in developing a multifunctional sensor. The hysteretic behavior of the sensor showed an acceptable value of hysteresis (∼12%). The response and recovery time for were found to be 13–15 seconds. The comparison given in Table 1 with previous studies shows that the sensing properties of the sensor have been significantly enhanced.

## Figures and Tables

**Table 1.** Comparison of sensitivity and bandwidth.

**Material**	**Sensitivity**	**Bandwidth**
PEPC+Cu_2_O [1]	0.83 pF/%RH	36–86%RH
NiPC [15]	8.93 pF/%RH	35–90%RH
PEPC+NiPc+ZnO [16]	12.2 pF/%RH	50–95%RH
PEPC+NiPC+ Cu_2_O [present work]	31.6 pF/%RH	40–100%RH
